# Comparing basal dendrite branches in human and mouse hippocampal CA1 pyramidal neurons with Bayesian networks

**DOI:** 10.1038/s41598-020-73617-9

**Published:** 2020-10-29

**Authors:** Bojan Mihaljević, Pedro Larrañaga, Ruth Benavides-Piccione, Javier DeFelipe, Concha Bielza

**Affiliations:** 1 Computational Intelligence Group, Departamento de Inteligencia Artificial, Universidad Politécnica de Madrid, Boadilla del Monte, 28660 Madrid, Spain; 2 Laboratorio Cajal de Circuitos Corticales, Universidad Politécnica de Madrid and Instituto Cajal (CSIC), Pozuelo de Alarcón, 28223 Madrid, Spain

**Keywords:** Cellular neuroscience, Computational neuroscience

## Abstract

Pyramidal neurons are the most common cell type in the cerebral cortex. Understanding how they differ between species is a key challenge in neuroscience. A recent study provided a unique set of human and mouse pyramidal neurons of the CA1 region of the hippocampus, and used it to compare the morphology of apical and basal dendritic branches of the two species. The study found inter-species differences in the magnitude of the morphometrics and similarities regarding their variation with respect to morphological determinants such as branch type and branch order. We use the same data set to perform additional comparisons of basal dendrites. In order to isolate the heterogeneity due to intrinsic differences between species from the heterogeneity due to differences in morphological determinants, we fit multivariate models over the morphometrics and the determinants. In particular, we use conditional linear Gaussian Bayesian networks, which provide a concise graphical representation of the independencies and correlations among the variables. We also extend the previous study by considering additional morphometrics and by formally testing whether a morphometric increases or decreases with the distance from the soma. This study introduces a multivariate methodology for inter-species comparison of morphology.

## Introduction

Pyramidal neurons are the main cell type in the mammalian cortex and a key challenge is to understand how they differ across species^1–5^. They are often compared in terms of their dendritic morphology, as it directly influences neuronal computation^6–10^. Dendritic morphology, however, varies across layers and cortical regions^4,11–15^, especially in primates^1,2^, and it is thus important to compare neurons from regions that are homologous between species. One such region is the hippocampus, considered one of the most evolutionarily conserved archicortical regions^16^. Within the hippocampus, of special interest are the CA1 pyramidal neurons cells, as their properties have been extensively studied^8^.

**Figure 1:**
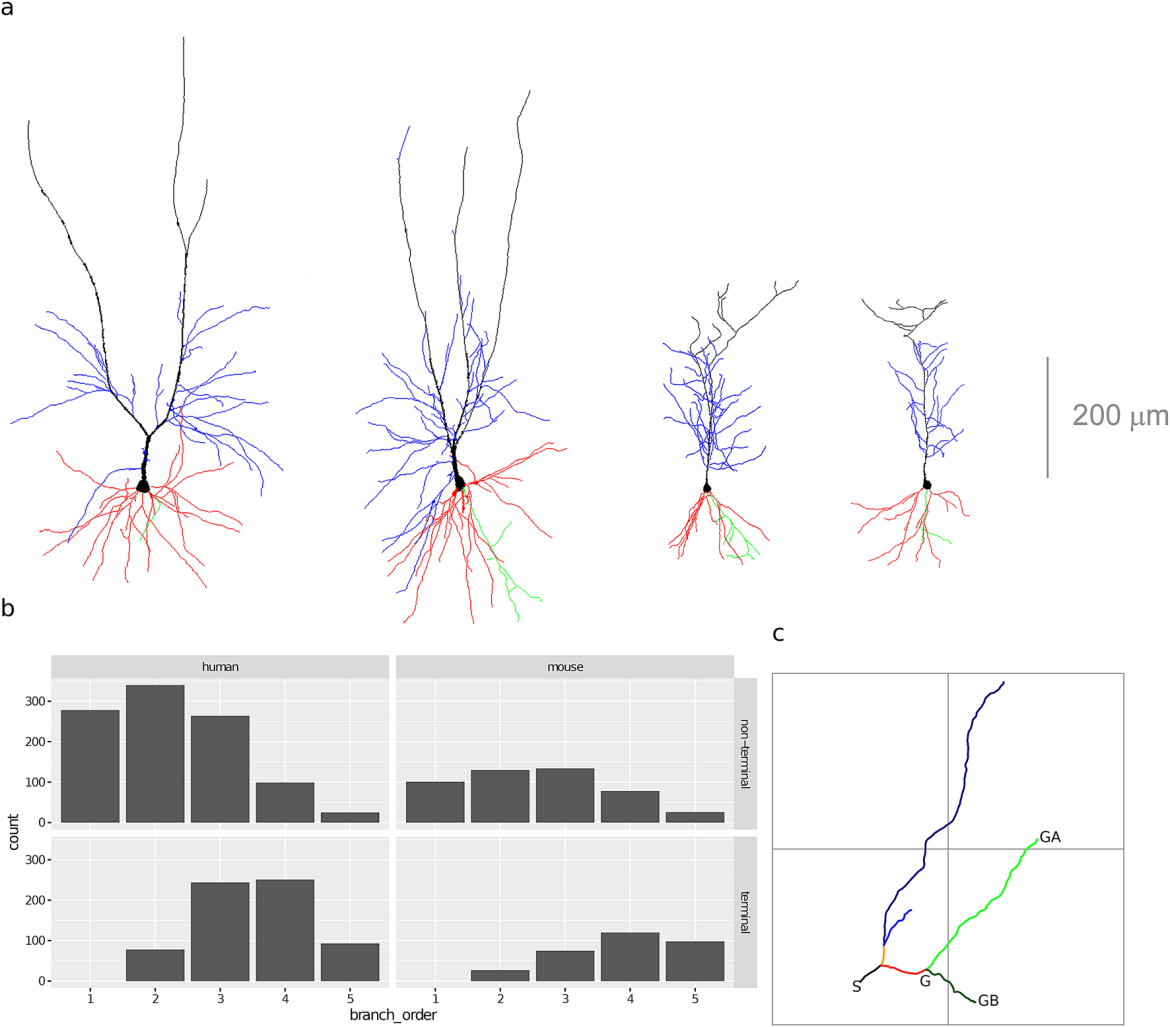
(**a**) Two human (left) and mouse (right) pyramidal cells’. Basal arbor is shown in red; main apical dendrite in black; apical collateral dendrites in blue, and axons in green (adapted from Ref.^20^). (**b**) Branch frequencies per species, branch type, and branching order. (**c**) An illustration of the computed morphometrics and morphological determinants. A branch (or dendritic segment) is defined as a sequence of cylinders between consecutive bifurcation points or between a bifurcation point and a terminal point (each branch shown with a different color). Branch orders (variable branch_order) are computed centrifugally, with branch order 1 corresponding to branches emanating from the soma (the black branch). The length (length) of a branch is the sum of the lengths of its cylinders. The average diameter (diameter) of a branch is the weighted average of the diameters of the branches’ cylinders. There are two types of branches (variable branch_type): non-terminal branches (black, red and orange branches) and terminal. The distance from the soma (distance) is the length of the straight line from the dendrites’ insertion point into the soma up to the starting point of a branch (e.g., for the green branches, this is the distance between S and G). Variables distance, length, and diameter are all measured in micrometers (µm). tortuosity describes dendritic meandering and is defined as the ratio of length and the length of the straight line between the beginning and the end of a branch; it is a unitless quantity and tortuosity $= 1$ denotes a perfectly straight branch whereas increasing values denote more tortuous branches. Remote bifurcation angle (bifurcation_angle) is the shortest planar angle between the vectors from the bifurcation to the endings of the daughter branches; for the red branch, it is the smaller angle between points GA, G, and GB. Remote tilt angle (tilt_angle) is the smallest angle between the branch director vector and the vectors from the bifurcation to the endings of the daughter branches. bifurcation_angle and tilt_angle are measured in radians. bifurcation_angle and length are undefined for terminal branches, as they do not bifurcate. The taper rate is ratio of the difference between the diameter of the first and last point of a branch and the diameter of the first point.

The dendritic arbor of a pyramidal neuron has two clearly distinct domains: the basal and the apical dendrites (see Fig. 1a). A large apical dendrite grows from the upper pole of the soma and bifurcates distally giving rise to the apical tuft, while short basal dendrites grow from the base of the soma. These two domains differ in terms of morphology, afferent connections, and ion channel distributions. In general, distant apical dendrites of CA1 pyramidal neurons receive input from the entorhinal cortex and the thalamic nucleus reuniens^17^ while their basal and proximal apical dendrites receive input primarily from CA3 cells via the Schaffer collaterals, with distant CA3 neurons projecting mainly to apical dendrites and CA3 neurons closer to CA1 projecting more to basal dendrites^18,19^. The two domains are separated in the mouse CA1 region, with the basal dendrites located in the stratum oriens and the apical dendrites in the stratum radiatum. In the human, on the other hand, they are intermixed in the pyramidal cell layer of CA1. While the differences between basal and apical dendrites suggest that they have distinct functions, the degree of such difference remains unclear^8^.

A recent study^20^ (henceforth ‘the previous study’) compared morphologies of pyramidal cells’ dendrites from the hippocampus CA1 area of human and mouse brains, using the first data set of digitally reconstructed morphologies of human CA1 pyramidal cells. Since the dendritic trees in the sample were incompletely reconstructed, the authors mainly focused on four branch-level morphometrics—branch length, surface, volume and (average) diameter—as they can be reliably quantified from incomplete dendritic trees, although they complemented their analysis with a number of whole-arbor metrics such as the Sholl analysis. The authors found that the two species had characteristic cell morphologies. In particular, human neurons were larger, more complex and their dendritic and axonal branches were longer and thicker than those of the mouse. Human terminal branches were also non-proportionally longer than their non-terminal branches, showing that the human neurons were not scaled versions of mouse neurons. There were, nonetheless, similarities regarding how the morphometrics varied with respect to morphological determinants such as branch type (whether a branch is terminal or not) and branching order. For example, terminal branches were longer than non-terminal ones in both species, as previously noted in neocortical pyramidal neurons^21^, while the diameter of terminal branches was constant across branch orders in both species.

Comparing morphometrics between species is not straightforward because they can vary with respect to determinants such as branch type and branch order. The authors of the previous study accounted for the determinants by splitting the branches according to determinants, that is into terminal and non-terminal ones and further down according to branch order, and then testing hypotheses of location difference (Kruskal–Wallis test) between pairs of subsets of branches (e.g., mouse terminal branches of branch order two are as long as human terminal branches of the same branch order). With two species, two branch types and five branch orders, we have $n = 2 \times 2 \times 5 = 20$ subsets of branches and $\frac{n(n - 1)}{2} = 190$ pairwise tests. This many tests might be hard to summarize while the splitting of the data into many subsets may result in low sample sizes.

Instead of directly splitting the branches according to the determinants and running multiple tests, our approach is to first learn from data a multivariate statistical model—a Bayesian network (BN)^22–24^—over the morphometrics and the morphological determinants. This simplifies location difference testing by identifying the probabilistic relationships among morphometrics and determinants. Indeed, finding that a variable is independent from the species variable in a BN amounts to not rejecting the hypothesis of equal location. Finding that a morphometric is dependent on the species variable in the BN means that it is likely that location differs yet this requires a posterior test in order to confirm it; namely, independence is rejected even with identical means if the variances differ between species. Importantly, this posterior location test is informed by the BN as it tells us which determinants we need to take into account. Thus, if the morphometric is independent of a determinant then we may safely ignore it and not split our data according to it, resulting in less tests with larger sample sizes.

The uncovered probabilistic relationships among morphometrics and determinants can be informative beyond location difference tests. The per-species BNs provides concise graphical representations of the probabilistic relationships among morphometrics and determinants and can be easily compared between species. For example, the correlations with the ‘distance from the soma‘ variable can tell us whether, and to what extent, a variable increases, decreases or stays constant with respect to the distance, providing something similar to a Sholl analysis. The BNs also allow us to distinguish among inter-species differences that may be explained in terms of other morphometrics and/or other determinants from those that cannot.

Thus, in this paper, we analyze the human and mouse hippocampal CA1 pyramidal cells obtained in the previous study^20^ with BNs. As in the previous study, we focus on branch-level morphometrics and, besides branch length and average branch diameter, we consider four additional branch-level morphometrics: tortuosity, bifurcation angles and tilt angles, and taper. We study how these morphometrics vary between species and according to three morphological determinants—branch type, branch order, and distance from the soma. We learn from data BN models for the joint distribution over the morphometrics and the determinants. In particular, we use conditional linear Gaussian Bayesian networks (CLGBNs), a distribution family that allows us to model both discrete and continuous variables. The conditional regression models contained within the CLGBNs allow us to identify the magnitude and the sign of correlations.

This study extends Ref.^20^ in a number of ways. First, we propose using BNs to summarize and simplify testing for location differences between species. The per-species and per-branch-type BNs provide models of branch-level morphology. We consider four morphometrics that were not covered in the previous study and perform Kruskal-Wallis tests of inter-species difference for them. We also formally test whether a morphometric increases, decreases or stays constant with the distance from the soma, by assessing linear correlation; while Ref.^20^ reported such observations they were based on hypotheses of equal medians across branch orders and thus could not establish the sign nor the magnitude of the effect. Based on the BNs, we provide possible explanations of some of the observed differences. For example, the higher taper of human branches could be explained in terms of their higher length alone and thus may simply be a consequence of this difference in length, rather than an intrinsic difference between human and mouse pyramidal neurons. Furthermore, human non-terminal branches were less tortuous than mouse ones independently of their length, thus possibly suggesting that human non-terminal branches are intrinsically less tortuosity than mouse ones.

We limit our analysis to the somewhat simpler case of basal dendrites, leaving apical dendrites for future work. Indeed, apical and basal dendrites’ morphology is usually modeled separately^25^. Even after accounting for differences between the main tuft and the obliques, as well as between the distal and proximal portion, the apical tree is more difficult to model than the basal one^26^. We learn BNs from three different subsets of our data: (a) from terminal branches alone; (b) from non-terminal branches alone; and (c) from both terminal and non-terminal branches. With (a) and (b) we learn models that are specific to each branch type whereas (c) allows us to study the differences between terminal and non-terminal branches. For data subsets (a) and (b) (c)) we learn a separate BN model for each species, whereas in (a) and (b) we also learn a combined BN model for both species. The combined ones allows us to identify the independencies and correlations with the species variable.

## Methods

### Data

All neuron morphology reconstructions were obtained by the authors of Ref.^20^ by intracellular injections in the CA1 region of the hippocampus. There were 54 human neurons, obtained at autopsy from two donors both no apparent neurological alterations (a male aged 45 and a female aged 53) within a postmortem interval of 2–3 h. Upon removal, the brains were immersed in cold 4% paraformaldehyde in 0.1 M phosphate buffer, pH 7.4 (PB). There were 50 mouse neurons, obtained from nine C57BL/6 adult (8-week-old) male mice. All animals were overdosed with sodium pentobarbital and perfused though the heart with phosphate-buffered saline (0.1 M PBS) followed by 4% paraformaldehyde in PB. 3D coordinates of the dendritic morphology were extracted using Neurolucida 360 (MicroBrightfield, VT, USA; see Fig. 1a). Since intracellular injections of the cells were performed in coronal sections, the part of the dendritic arbor nearest to the surface of the slice from which the cell soma was injected (typically at a depth of $\sim 30$ µm from the surface) was lost. Thus, some branches were not fully included within a section and were therefore incompletely reconstructed. For further details on tissue reconstruction, intracellular injections, immunocytochemistry, and cell reconstruction, see Ref.^20^.

We only considered branches from branch order 1 to branch order 5, as only 4% of the branches corresponded to higher branching orders. We omitted from our analysis 23 branches that had a single child branch, as well as one trifurcating branch, because angles are only defined for branches that bifurcate (i.e., have exactly two child branches); we kept the descendants of these branches. We omitted all incomplete branches from our analysis (all incomplete branches were labeled as terminal).

Our final sample consisted of 2462 branches, 1672 from 54 human neurons and 790 branches from 50 mouse neurons (see Fig. 1b). There were 136 (mean) $\pm 95$ (standard deviation) branches per each combination of species, branch type, and branching order (not considering branch order 1 for terminal branches, as there were none of this order). There were more human than mouse branches (1672 and 789, respectively) and more non-terminal than terminal branches (1475 and 986, respectively). In both species, roughly 60% of the branches were non-terminal, due to the removal of incomplete terminal branches, as half the branches of a fully reconstructed bifurcating tree are necessarily terminal and the other half non-terminal. A possible artifact of incomplete reconstruction was the higher proportion of order 4 and 5 terminal branches in the mouse data set (68%) than in the human one (52%; Fig. 1b). Namely, roughly half of the reconstructed human branches at order 5 were incomplete whereas that fraction was small for the mouse (see Fig. S3 in the Supplementary Information). This suggests that BO ought to be taken into account.

### Morphometrics

Because some branches were incompletely reconstructed, we did not use morphometrics that depend on the arbor being complete, such as total arbor length or topological variables such as partition asymmetry. Instead, we focused on sixbranch-level morphometrics (see Fig. 1c). Namely, we computed the branch length (length); remote bifurcation angle (bifurcation_angle); remote tilt angle (tilt_angle); average branch diameter (diameter); branch tortuosity (tortuosity ); and Hillman’s taper rate (taper). We considered one continuous morphological determinant, namely, the Euclidean distance from the branch starting point to the soma (distance), and three discrete ones, namely: species (species), (centrifugal) branch order (branch_order), and branch type (i.e., whether terminal or non terminal; branch_type). Note that bifurcation angles are only defined for non-terminal branches. While the previous study analyzed branch surface area and branch volume, in addition to length and diameter, we omitted these variables as they were deterministic functions of diameter and length and thus any differences between species would be implied by differences in diameter and length. We computed all morphometrics and determinants with the open-source NeuroSTR library (https://computationalintelligencegroup.github.io/neurostr/).

### Bayesian networks

A Bayesian network (BN)^22^ $\mathcal {B}$ allows us to compactly encode a joint probability distribution over a vector of *n* random variables **X** by exploiting conditional independencies among triplets of sets of variables in **X** (e.g., *X* is independent of *Y* given *Z*). A BN consists of a directed acyclic graph (DAG) $\mathcal {G}$ and a set of parameters $\varvec{ \theta }$ ($\mathcal {B} = (\mathcal {G}, \varvec{ \theta })$). The vertices (i.e., nodes) of $\mathcal {G}$ correspond to the variables in **X** while its directed edges (i.e., arcs) encode the conditional independencies among **X**. A joint probability distribution $P_{\mathcal {G}}(\mathbf {x})$ encoded by $\mathcal {B}$, where $\mathbf {x}$ is an assignment to **X**, factorizes as a product of local conditional distributions,

$$\begin{aligned} P_{\mathcal {G}}(\mathbf {x})= \prod _{i=1}^{n} P_{\mathcal {G}}(x_i \mid \mathbf {pa}_{\mathcal {G}}(x_i)), \end{aligned}$$

where $\mathbf {pa}_{\mathcal {G}}(x_i)$ is an assignment to variables $\mathbf {Pa}_{\mathcal {G}}(X_i)$, the set of parents of $X_i$ in **X** according to $\mathcal {G}$ (for a continuous variable, a probability mass function $P_{\mathcal {G}}(X_i)$ is replaced with a density function $f_{\mathcal {G}}(X_i)$). $\mathcal {G}$ induces conditional independence constraints for $P_{\mathcal {G}}(\cdot )$, derivable from the basic constraints that each $X_i$ is independent of its non-descendents in $\mathcal {G}$ given $\mathbf {Pa}_{\mathcal {G}}(X_i)$. For example, for any pair of variables *X*, *Y* in **X** that are not connected by an arc in $\mathcal {G}$ there exists a set of variables $\mathbf {Z}$ in $\mathbf{X}$ (disjoint from $\{X\}$ and $\{Y\}$) such that *X* and *Y* are independent conditionally on $\mathbf {Z}$ (i.e., $P_{\mathcal {G}}(X,Y \mid \mathbf {Z})$ = $P_{\mathcal {G}}(X \mid \mathbf {Z})$ $P_{\mathcal {G}}(Y \mid \mathbf {Z})$). Similarly, for any pair of variables *X*, *Y* in **X** that are connected by an arc in $\mathcal {G}$ there is no set $\mathbf {Z}$ such that *X* and *Y* are independent conditionally on $\mathbf {Z}$. These constraints extend to nodes not connected by an arc in $\mathcal {G}$ and the structure $\mathcal {G}$ thus lets us identify conditional independence relationships among any triplet of sets of variables *X*, *Y*, and $\mathbf {Z}$ in **X**. For example, in the DAG $X \rightarrow Y \rightarrow Z$ we only have one independence: *X* is independent of *Z* conditional on *Y*; *X* and *Y*, *X* and *Z*, and *Y* and *Z* are not marginally independent.

The parameters $\varvec{ \theta }$ specify the local conditional distributions (densities) $P_{\mathcal {G}}(X_i \mid \mathbf {pa}_{\mathcal {G}}(x_i))$ ($f_{\mathcal {G}}(x_i \mid \mathbf {pa}_{\mathcal {G}}(x_i))$) for each variable $X_i$. When **X** contains both discrete and continuous variables, as in our case, a common approach is to let $P_{\mathcal {G}}(\mathbf {x})$ be a conditional linear Gaussian distribution (CLG)^27^. The CLG only allows discrete parents in $\mathcal {G}$ for the discrete variables $\mathbf {D}$ in **X** and therefore the local conditional distribution of each discrete variable $D_i$ in $\mathbf {D}$ is a categorical distribution. The local conditional density $f_{\mathcal {G}}(g_i \mid \mathbf {pa}_{\mathcal {G}}(g_i))$ for a continuous variable $G_i$, in the set of continuous variables $\mathbf {G}$, is

$$\begin{aligned} f_{\mathcal {G}}(g_i \mid \mathbf {pa}_{\mathcal {G}}(g_i)) = f_{\mathcal {G}}(g_i \mid \mathbf {pa}_{\mathcal {G}}^{d}(g_i), \mathbf {pa}_{\mathcal {G}}^{g}(g_i)) = \mathcal {N}(g_i; \beta _0^{(\mathbf {pa}_{\mathcal {G}}^{d}(g_i))} + \varvec{\beta }^{(\mathbf {pa}_{\mathcal {G}}^{d}(g_i))T} \mathbf {pa}_{\mathcal {G}}^{g}(g_i), \sigma ^{(\mathbf {pa}_{\mathcal {G}}^{d}(g_i))2}_i), \end{aligned}$$

where $\mathbf {pa}_{\mathcal {G}}^{d}(g_i)$ is an assignment to the discrete parents of $G_i$ and $\mathbf {pa}_{\mathcal {G}}^{g}(g_i)$ an assignment to the continuous parents of $G_i$. There is thus a different vector of coefficients $\left( \varvec{\beta }, \sigma ^2\right)$ for each assignment $\mathbf {pa}_{\mathcal {G}}^{d}(g_i)$ and indeed the conditional density $f(\mathbf {g}\mid \mathbf {d})$ is a multivariate normal. $P_{\mathcal {G}}(\mathbf {x})$ is a mixture of multivariate normal distributions over $\mathbf {G}$, with one component for each instantiation $\mathbf {d}$ of $\mathbf {D}$.

### Learning Bayesian networks from data

Learning a Bayesian network $\mathcal {B}$ from a data set $\mathcal {D} = \{ \mathbf {x}^{1}, \ldots , \mathbf {x}^{N} \}$ of $N$ observations of **X** involves two steps: (a) learning the DAG $\mathcal {G}$; and (b) learning $\theta$, the parameters of the local conditional distributions. There are two main approaches to learning $\mathcal {G}$ from $\mathcal {D}$^22^: (a) by testing for conditional independence among triplets of sets of variables (the *constraint-based* approach); and (b) by searching the space of DAGs in order to optimize a score such as penalized likelihood (the *score-based* approach). While seemingly very different, conditional independence tests and network scores are related statistical criteria^28^. For example, when considering whether to include the arc $Y \rightarrow X$ into a graph $\mathcal {G}$, the likelihood-ratio test of conditional independence of *X* and *Y* given $\mathbf {Pa}_{\mathcal {G}}(X)$ and the Bayesian information criterion^29^ (BIC) score are both functions of $\log \frac{P\left( X | \mathbf {Pa}_{\mathcal {G}}(X), Y\right) }{P\left( X | \mathbf {Pa}_{\mathcal {G}}(X) \right) }$. They differ in computing the threshold for determining independence: the former relies on the distribution of the statistic under the null model (i.e., conditional independence) whereas the latter is based on an approximation to the Bayes factor between the null and alternative models. Besides using different criteria, the constraint-based and score-based approaches also differ in model search, that is, in terms of the sets *X*, *Y*, and $\mathbf {Z}$ that they choose to test conditional independence for. The score-based approaches tend to be more robust^22^, as they may reconsider previous steps in the search by removing or reversing previously added arcs. A typical score-based search algorithm is hill climbing, a local search which starts from some initial DAG $\mathcal {G}$ and greedily adds, removes or reverses arcs as long as that improves the score of the DAG. While classical hypothesis testing of conditional independence might be more in line with previous work on our data set^20^, score-based learning allows for more robust algorithms while still using sound statistical criteria for independence testing. We thus used the latter approach in this paper.

### Location and goodness-of-fit tests

We complement the previous study^20^ with location tests for tortuosity , bifurcation_angle, tilt_angle, and taper, morphometrics that were not considered in that paper. Although t-tests and the ANOVA fit naturally with conditional linear Gaussian Bayesian networks, we follow the previous study and apply the Kruskal–Wallis (KW) rank sum tests. We test our assumptions that the morphometrics are normally distributed conditional on species, branch_type and branch_order (see “Assumptions” section) with the Kolmogorov–Smirnov (KS) test. We set $\alpha = 0.05$ as the significance level for all tests.

### Assumptions

By modelling a joint distribution $P(\mathbf {x})$ with a $P_{\mathcal {G}}(\mathbf {x})$ encoded with a CLGBN, we are assuming the following about $P(\mathbf {x})$: (a) all conditional independencies in $P_{\mathcal {G}}(\mathbf {x})$ are present in $P(\mathbf {x})$; (b) $f(\mathbf {g}\mid \mathbf {d})$ is a multivariate normal density for each $\mathbf {d}$; and (c) variables in $\mathbf {D}$ have no parents in $\mathbf {G}$ in the DAG of $P(\mathbf {x})$. In particular, (a) means that some dependencies implied by $P_{\mathcal {G}}(\mathbf {x})$ need not hold in $P(\mathbf {x})$. (b) states that for each combination of species, branch_type, and branch_order, the distribution over diameter, length, tortuosity, distance, bifurcation_angle, and tilt_angle is a multivariate normal. (b) in turn implies that: (i) the morphometrics marginally follow a Gaussian distribution; and (ii) the dependencies among them are linear. (i) is a common simplifying assumption while we consider (ii) to a reasonable assumption given that there are not many cases (136 (mean) ± 95 (standard deviation) branches per combination of type, species, and branching order, see “Data” section). In addition, violations of (b) do necessarily lead to wrong arcs in $\mathcal {G}$, and we are precisely interested in the learning structure of $\mathcal {G}$ rather than the accurate estimation of $P(\mathbf {x})$. Since 3 of our 9 variables are discrete, (c) means that a significant number of arcs in $\mathcal {G}$ might have forced directions, as species, branch_type, and branch_order can have no parents in $\mathcal {G}$.

### Settings

As mentioned above, we consider three basic settings for learning Bayesian networks: from terminal branches alone (“Terminal branches” section); from non-terminal branches alone (“Non-terminal branches” section); and from both terminal and non-terminal branches (“All branches” section). In each setting, we learn three networks: one for the human branches; one for the mouse branches; and a combined model for branches of both species. Branching angles are only defined for non-terminal branches, and thus we omit them in models of terminal branches (“Terminal branches” section) as well as in combined models of terminal and non-terminal branches (“All branches” section). In “All branches” section we focus on the branch_type node and avoid analyzing dependencies among morphometrics, as branch-type specific dependencies can get obfuscated with the combined data from terminal and non-terminal branches that we use in “All branches” section.

All branches at branch order 1 had zero distance from the soma. To avoid a singular conditional Gaussian distribution for distance, we replaced these zeros by sampling from a normal distribution with mean 0 and standard deviation 0.00001.

We learned network structures by using the tabu algorithm^30^, implemented in the bnlearn^31^ R^32^ package, to optimize the BIC score. The tabu algorithm is a local search that efficiently allows for score-degrading operators by avoiding those that undo the effect of recently applied operators; we used a tabu list of size 30 and allowed for up to 30 iterations without improving network score. Since dependencies implies by the learned networks might be false positives (see assumption (a) in “Assumptions” section), we verify some of them with a posterior check. This consists in learning a Bayesian network by maximizing the BIC score over the variables in question only and verifying the dependence in that network.

## Results

We begin by assessing whether the conditional distributions of the morphometrics can be reasonably approximated with a Gaussian distribution when conditioned on species, branch_type and, optionally, branch_order (“Morphometrics’ conditional distributions” section). We then learn Bayesian networks from terminal branches (“Terminal branches” section), non-terminal branches (“Non-terminal branches” section), and both terminal branches and non-terminal branches (“All branches” section).

### Morphometrics’ conditional distributions

**Figure 2:**
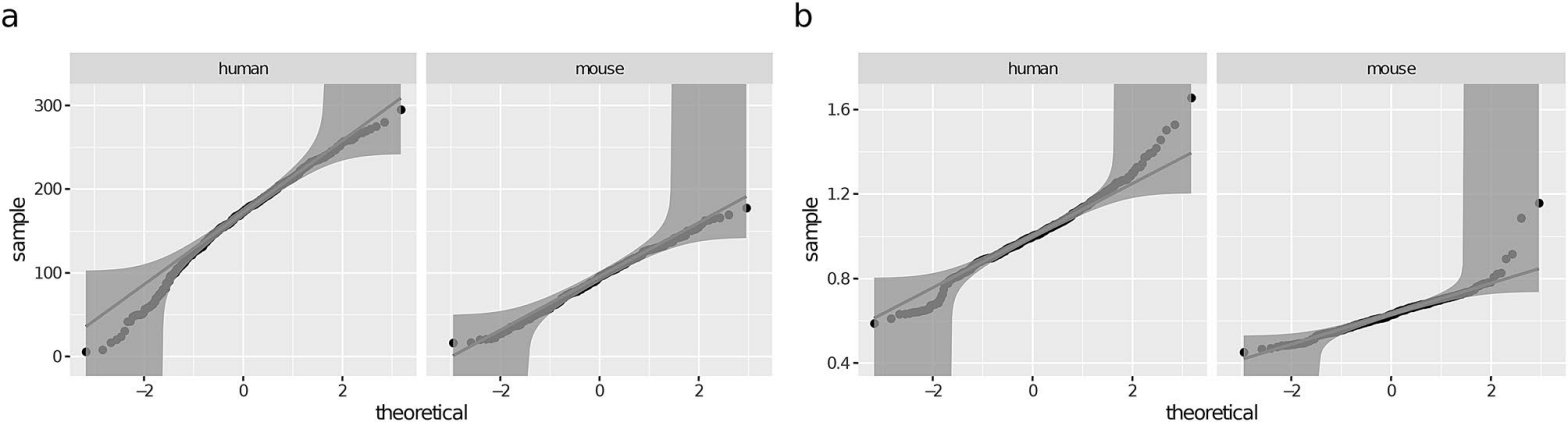
Q-Q plots of length (**a**) and diameter (**b**) of terminal branches conditional on species and branch_type. Normality is only rejected for human terminal branches, with a p-value of 0.04. The distribution of mouse length is especially close to the theoretical one (p-value 0.98). The plots show quantiles of the sample distributions on the *Y* axis and the quantiles of the standard normal distribution on the *X* axis. 95% KS confidence bands are shown; the hypothesis of normality is rejected at the 5% level if the sample does not fall completely within the bounds. Distributions that deviate little from normality have the points lying close to the straight line.

Considering the morphometrics’ distributions conditional on species and branch type (i.e., of the form $P(X \mid \texttt {species}{}, \texttt {branch\_type}{})$), the KS test did not reject normality (at the 0.05 significance level) for 6 out of 24 conditional distributions (see Table S1 in the Supplementary Information). For terminal branches, this included diameter for both species and length for the mouse (see Fig. 2). For non-terminals, it included bifurcation_angle for both species and tilt_angle for the mouse. When also conditioning on branch order (i.e., considering distributions of the form $P(X \mid \texttt {species}{}, \texttt {branch\_type}{}, \texttt {branch\_order}{})$), the KS test did not reject normality for 79 out of 108 conditional distributions (see Table S1 in the Supplementary Information). This included bifurcation_angle and tilt_angle regardless of species, branch type and branch order, as well as length and diameter of terminal branches and taper of non-terminal ones, for either species. On the other hand, the KS test rejected normality at most branch orders for the length of non-terminal branches and tortuosity of human branches. In summary, the conditional distributions of most morphometrics can be approximated with Gaussian distributions after accounting for branch_order.

### Terminal branches

**Figure 3:**
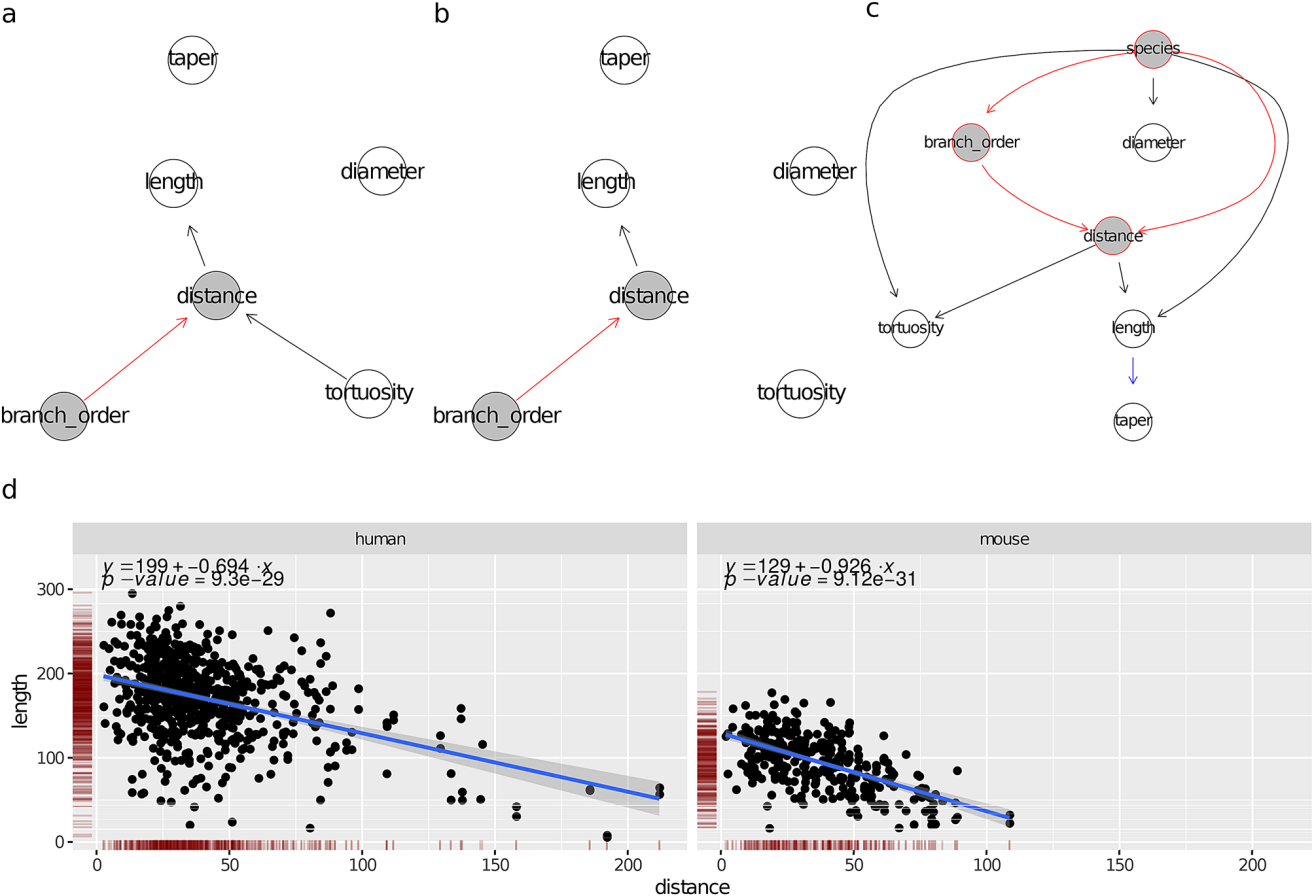
Bayesian networks learned from the terminal branches of human neurons (**a**), mouse neurons (**b**), and neurons of both species (**c**). Proximity between two nodes of a graph is unrelated to the strength of their correlation. The nodes of the morphological determinants are shaded in grey with red borders. An arc between two morphological determinants is depicted in red; an arc between a determinant and a morphometric in black. (**d**) Scatter plots depicting the conditional distribution of length on distance, for the human (left) and for the mouse (right). The linear regression line shows the mean of the fitted conditional distribution of length as a function of distance, with its formula given in the top part of the panel, with *y* standing for length and *x* for distance. The band shows a 95% confidence interval around the mean.

The learned human and mouse BNs (Fig. 3a,b, respectively) uncovered a set of independencies among the variables. For example, diameter and taper were independent of all determinants and morphometrics, including length, in both species, as their nodes were disconnected from the rest of the graph in Fig. 3a,b. tortuosity and length were independent in the mouse BN and a posterior analysis found them independent in human branches as well, although the human BN did not imply this independence (due to the path tortuosity$\rightarrow$ distance $\rightarrow$ length).

**Figure 4:**
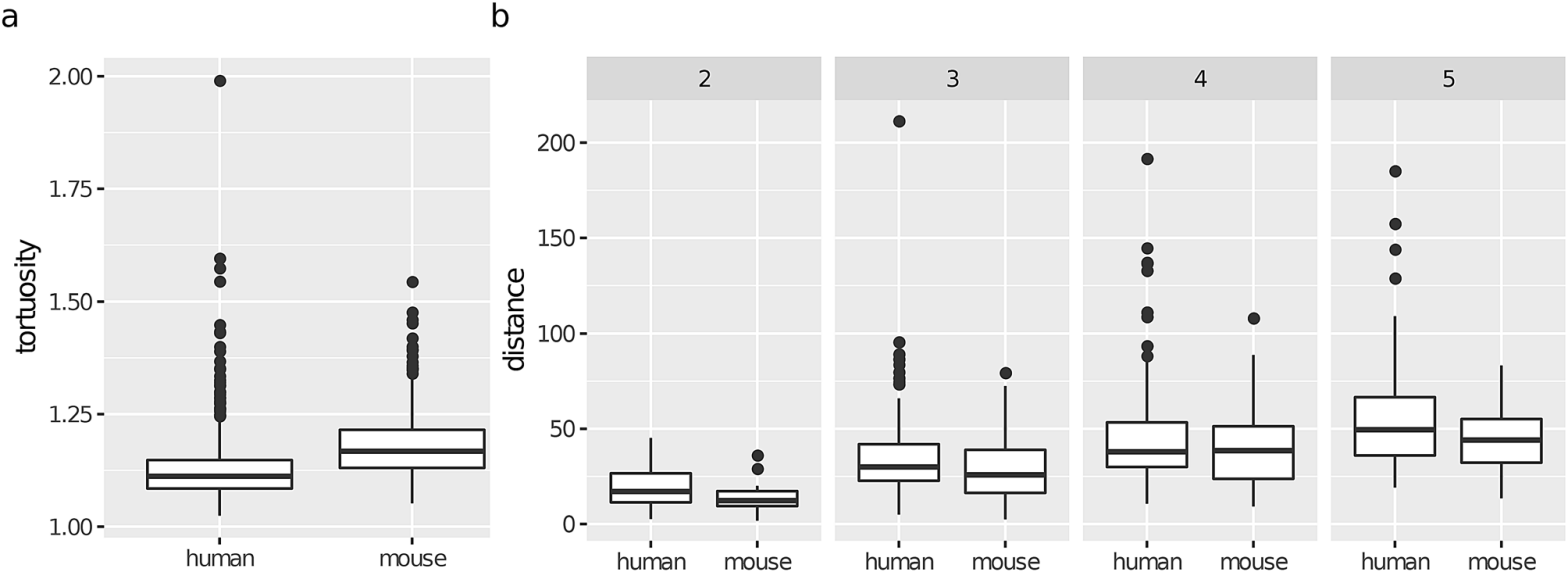
Differences in magnitude of tortuosity (**a**) and distance (**b**) of terminal branches between the species. Human terminal branches were less tortuous than mouse terminal branches (KW p-value $< 0.0001$ for tortuosity). Marginally, they were not located further away from the soma than mouse ones (p-value 0.26 for distance) yet, for branch orders 2, 3 and 5, they were (p-values 0.0096, 0.0091, 0.0897, and 0.0398 for distance at branch orders 2, 3, 4, and 5, respectively).

The correlations of the determinants (branch_order and distance) with the morphometrics were similar across species. Namely, length decreased with distance (see Fig. 3d), with a correlation coefficient $\rho = -\, 0.58$ for the mouse and $\rho = -\, 0.41$ for the human. In addition, length was independent of branch_order given distance for both species. On the other hand, as mentioned above, diameter was independent of both branch_order and distance. What differed between the species was that tortuosity slightly increased with distance in human cells ($\rho = 0.30$; note the tortuosity $\rightarrow$ distance arc) while in the mouse it stayed constant (no arcs in or out of tortuosity ). In summary, the effect of increasing distance was that length decreased, diameter and taper stayed constant, while tortuosity increased slightly in human branches yet stayed constant in the mouse ones. Naturally, distance itself increased with branch_order (note the branch_order $\rightarrow$ distance arc in both BNs; see also Fig. 4). Overall, the human and mouse BNs were similar, with the only structural difference being that the tortuosity $\rightarrow$ distance arc was present in the human BN yet not in the mouse BN.

The combined BN for terminal branches (Fig. 3c) shows that the determinants’ distributions varied between the human and mouse data sets, as there were arcs from the species node to both branch_order and distance. Indeed, there were proportionally more order 4 and 5 terminal branches in the mouse data set (68%) than in the human one (52%), which was likely an artifact of incomplete reconstruction rather than an actual difference in branching orders between the two species (“Data”’ section). On the other hand, a KW test found no difference (p-value of 0.26) in distance between the species. However, as Fig. 3c implies (both species and branch_order and parents of distance in the BN), we confirmed that human branches were located further away from the soma than mouse ones of the same order (Fig. 4b). This difference between species was obfuscated marginally by the higher proportion of high order branches in the mouse data set and was thus undetected by the KW test.

The combined BN for terminal branches also shows that the distributions of all morphometrics differed between species. In particular, human terminal branches were less tortuous (Fig. 4a) and tapered more than mouse terminal branches (Fig. S5 in the Supplementary Information); as reported by the previous study, they were also thicker and longer. Figure 3c also shows that these differences were not only due to differences in determinants between the two samples (e.g., proportionally more high order branches in the mouse), because the species node was not independent of any morphometric given any the determinants. Except for taper, these differences could also not be explained in terms of other morphometrics. For example, the lower tortuosity of human terminal branches was unrelated to their higher length, as tortuosity was independent of length given distance in the combined BN. These were, therefore, intrinsic differences between human and mouse terminal branches. The difference in taper, on the other hand, was not necessarily intrinsic, as taper was independent of the species node conditional on length. Namely, it may be that human neurons’ branches tapered more because they were longer. This positive correlation between length and taper was just below significance when considering human neurons alone and thus an arc between length and taper is missing in Fig. 3a, and just above significance when combining branches from the two species.

The difference in diameter was in fact unrelated to differences in the determinants, as diameter was independent of all other variables given species. The differences in length and tortuosity , on the other hand, could not be explained in terms of intrinsic inter-species differences alone, because length and tortuosity were not independent of the determinants given species (distance and species were parents of both tortuosity and length). Nonetheless, we found that the inter-species ratios of mean length and tortuosity were roughly constant across branch orders.

### Non-terminal branches

**Figure 5:**
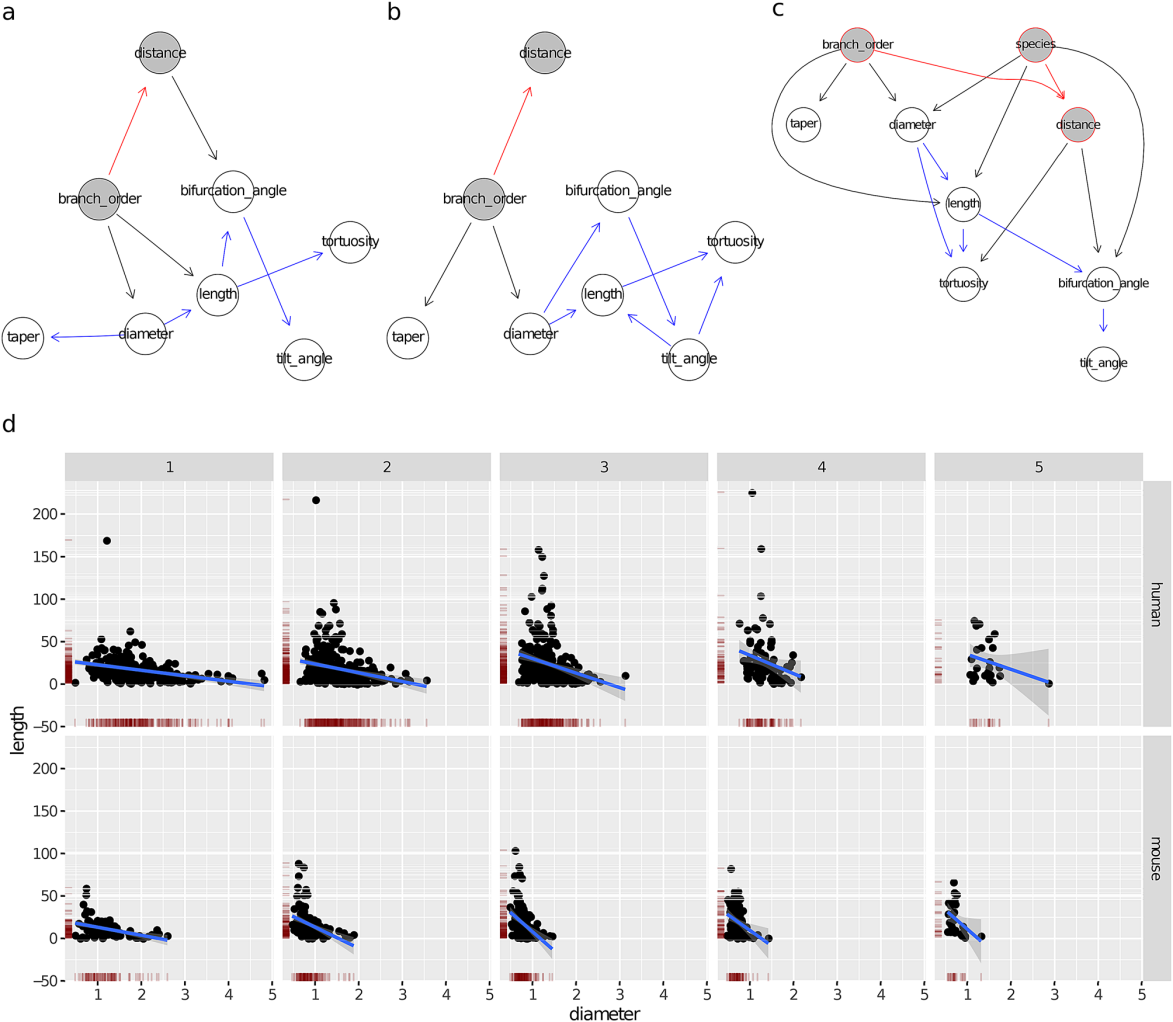
Bayesian networks learned from the non-terminal branches of human neurons (**a**), mouse neurons (**b**), and neurons of both species (**c**). Proximity between two nodes of a graph is unrelated to the strength of their correlation. The nodes of the morphological determinants are shaded in grey with red borders. An arc between two morphological determinants is depicted in red; an arc between two morphometrics in blue; an arc between a determinant and a morphometric in black. (**d**) Scatter plots depicting the conditional distribution of length on species, branch_order and distance. The linear regression line shows the mean of the fitted conditional distribution of length given distance, with a 95% confidence interval; there is one regression line for each combination of branch_order and species. Since length is independent of branch_order given diameter in (**b**), the regression lines are roughly parallel across branch orders for the mouse.

The human BN (Fig. 5a) and the mouse BN (Fig. 5b) uncovered a number of conditional independencies among the variables. In the mouse BN, for example, all morphometrics except for taper were independent of the determinants—branch_order and distance—given diameter. This suggests a simple ‘localized’ model of morphology that depends on the determinants only through the diameter; see “Discussion” section below. Branch taper varied with branch_order but, given branch_order, it was independent of all variables in both BNs.

The correlations between the determinants (branch_order and distance) and the morphometrics were similar between the two species, with same-sign correlation coefficients for all morphometrics (see Fig. 1 in the Supplementary Information). As expected, diameter decreased with distance ($\rho = -\,0.35$ for the human and $\rho = -\,0.47$ for the mouse) while length slightly increased. bifurcation_angle and tilt_angle decreased slightly; this is consistent with the previous finding in mouse neocortical cells showing that the initial bifurcation angles of a neuron are wider than those at higher branch orders^33^. On the other hand, tortuosity and taper stayed constant regardless of distance. Unlike the terminal branches, taper varied with branch_order, yet not with distance, in both species. The relationship was non-linear: taper increased when going from order 1 to order 2 and then decreased at order 3 (see Fig. S4l in the Supplementary Information); this increase/decrease trend was the same in the two species, except at branch order 5. After accounting for branch_order, distance was uninformative for all variables in the mouse BN and all but bifurcation_angle and tilt_angle in the human BN. Thus, branch_order was the more informative determinant.

A number of correlations among morphometrics were similar across the species. In particular, diameter and length were negatively correlated (see Fig. 5d), with the correlation coefficient varying across branch orders for the human yet not for the mouse (hence no branch_order $\rightarrow$ length arc in the mouse BN). tortuosity was positively correlated with length in both species, with the longer branches being more tortuous (see Fig. S2 in the Supplementary Information). bifurcation_angle increased slightly with diameter ($\rho = 0.11$ for the human and $\rho = 0.29$ for the mouse) and decreased slightly with length ($\rho = -\,0.17$ for the human and $\rho = -\,0.21$ for the mouse). tilt_angle was positively correlated with bifurcation_angle in both human ($\rho = 0.58$) and mouse ($\rho = 0.53$). Interestingly, taper was uncorrelated with length in both species, meaning that longer branches did not taper more than short ones.

**Figure 6:**
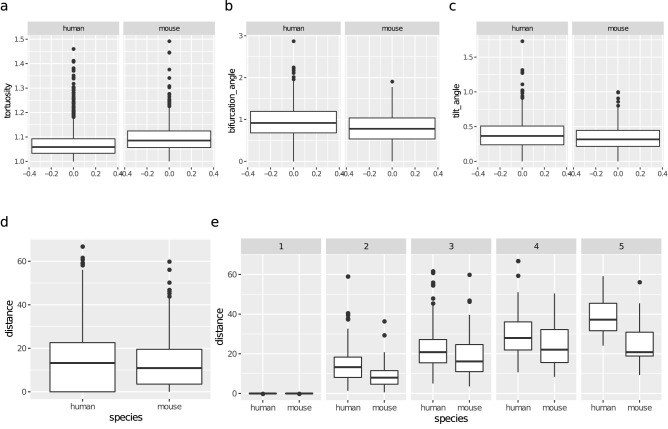
Differences in magnitude of variables of non-terminal branches between the species. Human non-terminal branches were less tortuous (**a**), and had larger bifurcating (**b**) and tilt angles (**c**) (KW p-values <0.0001). Marginally, human non-terminal branches were not located further away from the soma than mouse ones ((**d**); p-value 0.2267 for distance) yet (**e**), for all branch orders except for 1, they were (p-value of 0.3377 at branch order 1, 0.0003 at branch order 4, and $< 0.0001$ at branch orders 2, 3, and 5).

The combined BN for non-terminal branches (Fig. 5c) shows that the distribution of branch_order did not vary significantly between the mouse and human data sets (see also Fig. 1b) while the distribution of the distance determinant did. While there was no difference marginally (KW p-value of 0.2267) (Fig. 6d), human non-terminal branches were indeed located further away from the soma than mouse non-terminal branches of the same branching order (Fig. 6e).

Figure 5c also shows that the distributions of all morphometrics other than taper differed between species. In particular, we found that human non-terminal branches were less tortuous and had wider bifurcation and tilt angles than mouse ones (Fig. 6a–c); as reported by the previous study, they were also thicker and longer than those of mouse. However, the inter-species differences in length, bifurcation_angle, and tilt_angle were less pronounced or inexistent when conditioning on branch order. In particular, differences in length were insignificant at branch orders 2, 4, and 5; in bifurcation_angle at branch orders 4 and 5; while the differences in tilt_angle were only significant at branch order 2 (see Fig. S5 in the Supplementary Information). There was no such effect on diameter and tortuosity and they differed between species even after conditioning on branch_order. It is remarkable that mouse branches were more tortuous than human ones , since they are either shorter or of similar length (Fig. S4 in the Supplementary Information) and shorter branches tend to be more straight in both species. In particular, Fig. S2 in the Supplementary Informationshows that mouse branches were more tortuous than human ones of the same length. This either suggests an intrinsically lower tortuosity in human dendrites or an effect of diameter on tortuosity that is independent of length; indeed, tortuosity was independent of species given length, diameter, and distance in Fig. 5c. In summary, a genuine difference between species is that human dendrites are thicker; the differences in length, bifurcation_angle and tilt_angle vanished at most branch orders while the difference in tortuosity might simply be a consequence of differences in length, diameter, and distance. We note that the bifurcation angles in mouse neurons were notably lower than those reported for cortical neurons in the mouse^33^ at all branch orders (e.g., mean angle of 54.12° at branch order 1 versus 59.68° in M2 cortical pyramidal neurons, and higher in other areas considered).

### All branches

**Figure 7:**
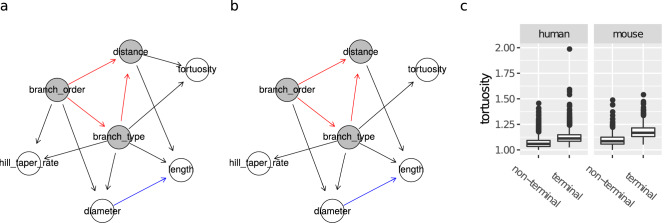
Bayesian networks learned from terminal and non-terminal branches of human data (**a**), mouse data (**b**), and differences in magnitude of tortuosity between terminal and non-terminal branches across species (**c**). Proximity between two nodes of a graph is unrelated to the strength of their correlation. The nodes of the morphological determinants are shaded in grey with red borders. An arc between two morphological determinants is depicted in red; an arc between two morphometrics in blue; an arc between a determinant and a morphometric in black.

The human (Fig. 7a) and mouse (Fig. 7b) BNs for all branches show that all variables varied with respect to branch_type. In particular, terminal branches were, in both species, more tortuous than non-terminal ones (KW p-values $< 0.0001$; see Fig. 7c). Terminal branches tapered more than non-terminal ones in the human; in the mouse they had wider range of taper values than non-terminal ones yet medians did not differ (Fig. S6 in the Supplementary Information). As reported by the previous study, terminal branches were also thinner and longer than non-terminal branches. The differences with respect to length, diameter, tortuosity, and taper between terminal and non-terminal branches could not be explained only in terms of the remaining determinants nor the remaining morphometrics, as branch_type was directly connected to all other variables in Fig. 7a,b. For example, the lower tortuosity of non-terminal branches could not be explained only by their shorter length, as tortuosity is not independent of branch_type given length in Fig. 7a nor in Fig. 7b. These seem to be, instead, intrinsic differences between terminal and non-terminal branches. The human and mouse BNs were similar, with the only structural difference being that the distance $\rightarrow$ tortuosity and branch_order $\rightarrow$ taper arcs were present in the human BN yet not in the mouse BN.

## Discussion

This paper introduced Bayesian networks (BNs) as a multivariate model for comparison between two samples—in this case, the neurons of two species—when one needs to control for discrete determinants. In terms of testing for equal location, one does not reject the null hypothesis for those variables that are independent of the species variable in a network (namely, taper in Fig. 5). For the remaining variables, the networks indicate which determinants can be omitted for posterior tests of equal location, thereby increasing their sample size; for example, we were able to ignore all determinants when testing for equality of terminal branches’ diameter, because diameter was conditionally independent of them in Fig. 3c. The BNs provided concise graphical representations of the branch-level morphology in terms of probabilistic relationships among morphometrics and determinants and thus allowed us to easily identify some differences and similarities between species. They also let us isolate intrinsic inter-species differences from the heterogeneity that is due to differences in the distribution of morphological determinants such as branch order and branch type, as well as to hypothesize regarding the explanations of the observed differences. The BNs thus provide a comprehensive approach to comparison between samples. While BNs have been previously used for modelling dendritic morphology, our study is the first to use conditional linear Gaussian Bayesian networks, allowing us to: (a) avoid transforming numerical variables into categorical ones, which would imply losing information and would make interpretation harder; and (b) quantify the marginal and partial correlation among variables.

The correlations among the morphometrics, as well as between the determinants and the morphometrics, were largely similar between species yet different between terminal and non-terminal branches of a species. Indeed, the terminal branches’ BNs (“Terminal branches” section) differed in only one arc while the non-terminal ones (“Non-terminal branches” section) had five arcs in common. The signs of the correlations coefficients were often the same in human and mouse BNs in both terminal and non-terminal branches.

Overall, a number of differences between terminal and non-terminal branches were consistent across species. First, terminal branches were more tortuous than non-terminal ones, and this was independent of them being longer. Second, branch length decreased with distance from the soma for terminal branches (Fig. 3d) whereas it slightly increased for non-terminal branches (see Fig. S1 in the Supplementary Information). Third, the models for terminal branches were simpler, with less arcs per node in the BNs (thus more independencies among variables) and the conditional distributions of length and diameter were well approximated by a normal distribution for any branching order in the terminal branches yet for relatively few in the non-terminal ones (see Table S2 in the Supplementary Information). Fourth, branch length decreased with the distance from soma in terminal branches while it slightly increased in non-terminal ones. Fifth, in terminal branches, the distance from soma was more informative about branch length than branch order, as length was independent of branch order given distance in both species (Fig. 3a,b), whereas in non-terminal branches distance was independent, conditional on branch order, of all morphometric in the mouse network (Fig. 5b) and of four out of six morphometrics in the human network (Fig. 5a). A possible explanation is that the wide range of distances within a branch order of terminal branches (up to 211.81 µm in Fig. 4), relative to the narrow range of distances for non-terminal branches (up to 66.93 µm in Fig. 6e), allows for variability in length within a branching order. Thus, when analysing terminal branches it is informative to consider the distance from the soma as complementary information to branch order whereas for non-terminal branches accounting for branch order alone may suffice.

Accounting for the spatial position of a branch—via its distance from the soma or branch order—was required in order to model the distribution of some morphometrics. Indeed, Ref.^26^ found that virtual dendrites models that ignored such information failed to replicate some features, such as topological asymmetry, of real dendrites. We found that, in non-terminal branches, spatial position affected the distribution of length, diameter and the angles (see Fig. S1 in the Supplementary Information). Interestingly, the morphometrics of mouse non-terminal branches were, with the exception of the taper rate, independent of the determinants given the diameter. This may suggest a growth model where the spatial position affects the diameter, which in turn largely determines the remaining morphometrics. Such a model would, however, be insufficient for human non-terminal branches, as both branch order and the distance from soma were still informative for length and the angles after accounting for diameter. Thus, the spatial position determinants, which might be capturing the effects of extra-cellular signaling pathways onto dendritic growth, might have a stronger effect in human cells than in mouse cells.

Such a diameter-based growth model would not apply, however, to terminal branches, as diameter was independent of spatial position; indeed, diameter was independent of all other variables in both species. The independence might be explained by Hillman’s model^34,35^ of neuronal growth. Namely, it states that the diameter determines whether a branch bifurcates, meaning that it directly affects its length and therefore makes the diameter and length variables correlated in non-terminal (i.e., bifurcating) branches. The absence of this branching effect of diameter in the terminal branches might then explain the independence of the diameter from the branch length variable, and perhaps with other variables as well. It is interesting, however, that branch length decreased with the distance from soma while the diameter did not, perhaps suggesting a different effect of extra-cellular signalling pathways on length and diameter. Unlike in the non-terminal branches, where longer branches were thinner and more tortuous than short ones, terminal branches’ length, diameter and tortuosity were basically uncorrelated. In addition, the (conditional) distributions of length and diameter were well approximated by Gaussians. This yields simple models of terminal branches’ morphology, with normally distributed variables and only the mean of branch length depending on spatial position. Interestingly, this simple model for terminal branches was largely consistent across the two species.

We found a number of inter-species differences in tortuosity, taper, and bifurcation and tilt angles. Human terminal dendritic branches were clearly less tortuous and had wider bifurcation and tilt angles than mouse terminal branches. While they also tapered more, the combined BN (Fig. 3c) shows that it might be a consequence of them being longer. Similarly, human non-terminal branches were less tortuous and had wider angles than mouse ones. Indeed, human non-terminal branches were less tortuous than mouse ones of the same length, suggesting either an intrinsically lower tortuosity or an effect of diameter on tortuosity. Overall, however, inter-species differences were less pronounced in non-terminal than in terminal branches, as already noted by the previous study and Ref.^15^ regarding the branch length of basal dendrites in temporal cortex pyramidal neurons of the human and the mouse. In particular, the differences in bifurcation and tilt angles, as well as the difference in branch length, were nonexistent at most branch orders (“Non-terminal branches” section). Since incomplete arbor reconstruction may have altered branch order distribution, it may have caused the marginally observed differences in bifurcation angles, tilt angles, and branch length, and these are thus possibly not genuine differences. In summary, human non-terminal branches were clearly less tortuous than mouse ones and, as identified by the previous study, they were thicker; the differences regarding the remaining morphometrics were less clear. The direct functional implications of many of these inter-species differences are not obvious, as much is still under research regarding how morphology affects function^34^. It is clear, however, that the intrinsic firing properties of pyramidal neurons vary considerably along with differences in dendritic structure and channel distributions, suggesting that different pyramidal neurons carry out specialized functions (reviewed in Ref.^8^). Indeed, previous studies have modeled human neuron morphological properties demonstrating the biophysical and computational distinctiveness of human cortical neurons (e.g. Ref.^36–39^). Certainly, larger angles allow human branches to occupy more volume and thus form more connections and reach further, and might be related to the differences in scale between the human and mouse brains (see also Ref.^40^). Further research may help to clarify these implications.

Provided that our assumptions hold (see “Assumptions” section), the learned BNs are faithful models of the branch-level morphologies for the two species. As such, they could be used for more purposes than comparison. For example, to generate synthetic branches^21^ or to perform probabilistic queries about the morphology. The models could be adapted to handle non-linear relationships, either by transforming variables (e.g., taking the logarithm) or by using kernel-based tests of conditional independence^41^.

## Supplementary information

Supplementary Information.

## Data Availability

The data analyzed in the current study are being published at a public repository. In the mean time, they are available from the corresponding author upon request.

## References

[1] Luebke JI (2017). Pyramidal neurons are not generalizable building blocks of cortical networks. Front. Neuroanat..

[2] Gilman JP, Medalla M, Luebke JI (2017). Area-specific features of pyramidal neurons: A comparative study in mouse and rhesus monkey. Cereb. Cortex.

[3] Elston GN, Benavides-Piccione R, DeFelipe J (2001). The pyramidal cell in cognition: A comparative study in human and monkey. J. Neurosci..

[4] Mohan H (2015). Dendritic and axonal architecture of individual pyramidal neurons across layers of adult human neocortex. Cereb. Cortex.

[5] Bianchi S (2013). Dendritic morphology of pyramidal neurons in the chimpanzee neocortex: Regional specializations and comparison to humans. Cereb. Cortex.

[6] Segev I, London M (2000). Untangling dendrites with quantitative models. Science.

[7] Häusser M, Spruston N, Stuart GJ (2000). Diversity and dynamics of dendritic signaling. Science.

[8] Spruston N (2008). Pyramidal neurons: Dendritic structure and synaptic integration. Nat. Rev. Neurosci..

[9] Mason A, Larkman A (1990). Correlations between morphology and electrophysiology of pyramidal neurons in slices of rat visual cortex. II. Electrophysiology. J. Neurosci..

[10] Mainen ZF, Sejnowski TJ (1996). Influence of dendritic structure on firing pattern in model neocortical neurons. Nature.

[11] Benavides-Piccione R, Ballesteros-Yáñez I, DeFelipe J, Yuste R (2002). Cortical area and species differences in dendritic spine morphology. J. Neurocytol..

[12] Benavides-Piccione R, Hamzei-Sichani F, Ballesteros-Yáñez I, DeFelipe J, Yuste R (2006). Dendritic size of pyramidal neurons differs among mouse cortical regions. Cereb. Cortex.

[13] Ballesteros-Yáñez I, Benavides-Piccione R, Bourgeois J, Changeux J, DeFelipe J (2010). Alterations of cortical pyramidal neurons in mice lacking high-affinity nicotinic receptors. Proc. Natl. Acad. Sci..

[14] Rojo C (2016). Laminar differences in dendritic structure of pyramidal neurons in the juvenile rat somatosensory cortex. Cereb. Cortex.

[15] Deitcher Y (2017). Comprehensive morpho-electrotonic analysis shows 2 distinct classes of l2 and l3 pyramidal neurons in human temporal cortex. Cereb. Cortex.

[16] Stephan, H. & Andy, O. J. The allocortex in primates. in *The Primate Brain*, vol. 1, 109–135 (Appleton Century Crofts, 1970).

[17] Andersen P, Morris R, Amaral D, Bliss T, OKeefe J (2006). The Hippocampus Book.

[18] Ishizuka N, Weber J, Amaral DG (1990). Organization of intrahippocampal projections originating from CA3 pyramidal cells in the rat. J. Compar. Neurol..

[19] Li X-G, Somogyi P, Ylinen A, Buzsáki G (1994). The hippocampal CA3 network: An in vivo intracellular labeling study. J. Compar. Neurol..

[20] Benavides-Piccione R (2020). Differential structure of hippocampal CA1 pyramidal neurons in the human and mouse. Cereb. Cortex.

[21] López-Cruz PL, Bielza C, Larrañaga P, Benavides-Piccione R, DeFelipe J (2011). Models and simulation of 3D neuronal dendritic trees using Bayesian networks. Neuroinformatics.

[22] Koller D, Friedman N (2009). Probabilistic Graphical Models: Principles and Techniques.

[23] Pearl J (1988). Probabilistic Reasoning in Intelligent Systems: Networks of Plausible Inference.

[24] Bielza C, Larrañaga P (2014). Bayesian networks in neuroscience: A survey. Front. Comput. Neurosci..

[25] Van Pelt J, Dityatev AE, Uylings HB (1997). Natural variability in the number of dendritic segments: Model-based inferences about branching during neurite outgrowth. J. Compar. Neurol..

[26] Donohue DE, Ascoli GA (2005). Local diameter fully constrains dendritic size in basal but not apical trees of CA1 pyramidal neurons. J. Comput. Neurosci..

[27] Lauritzen S, Wermuth N (1989). Graphical models for associations between variables, some of which are qualitative and some quantitative. Ann. Stat..

[28] Scutari M, Graafland CE, Gutiérrez JM (2019). Who learns better Bayesian network structures: Accuracy and speed of structure learning algorithms. Int. J. Approx. Reason..

[29] Schwarz G (1978). Estimating the dimension of a model. Ann. Stat..

[30] Glover F, Laguna M, Pardalos PM, Du D-Z, Graham RL (2013). Tabu search. Handbook of Combinatorial Optimization.

[31] Scutari M (2010). Learning Bayesian networks with the bnlearnR package. J. Stat. Softw..

[32] R Core Team. *R: A Language and Environment for Statistical Computing*. R Foundation for Statistical Computing (2015).

[33] Bielza C, Benavides-Piccione R, López-Cruz P, Larranaga P, DeFelipe J (2014). Branching angles of pyramidal cell dendrites follow common geometrical design principles in different cortical areas. Sci. Rep..

[34] Ascoli GA (1999). Progress and perspectives in computational neuroanatomy. Anat. Rec..

[35] Hillman D (1979). Neuronal shape parameters and substructures as a basis of neuronal form.

[36] Beaulieu-Laroche L (2018). Enhanced dendritic compartmentalization in human cortical neurons. Cell.

[37] Eyal G (2016). Unique membrane properties and enhanced signal processing in human neocortical neurons. eLife.

[38] Eyal G (2018). Human cortical pyramidal neurons: From spines to spikes via models. Front. Cell. Neurosci..

[39] Goriounova NA (2018). Large and fast human pyramidal neurons associate with intelligence. Elife.

[40] Fernandez-Gonzalez P (2017). Dendritic-branching angles of pyramidal neurons of the human cerebral cortex. Brain Struct. Funct..

[41] Zhang, K., Peters, J., Janzing, D. & Schölkopf, B. Kernel-based conditional independence test and application in causal discovery. *Uncertainty in Artificial Intelligence*, 804–813 (2011).

