# Supplementary material for: Comparing basal dendrite branches in human and mouse hippocampal CA1 pyramidal neurons with Bayesian networks
Source: Sci Rep. 2020 Oct 29;10:18592. doi: 10.1038/s41598-020-73617-9 (PMC7596062; doi:10.1038/s41598-020-73617-9)
Supplement: Supplementary file 1 — Supplementary Information. [file 41598_2020_73617_MOESM1_ESM.pdf]

Supplementary information: Comparing basal dendrite branches in human and mouse hippocampal CA1 pyramidal neurons with Bayesian networks

Bojan Mihaljević[1,*], Pedro Larrañaga[1], Ruth Benavides-Piccione[2], Javier DeFelipe[2], and Concha Bielza[1]

[1]Computational Intelligence Group, Departamento de Inteligencia Artificial, Universidad Politécnica de Madrid, Boadilla del Monte, 28660, Spain
[2]Laboratorio Cajal de Circuitos Corticales, Universidad Politécnica de Madrid and Instituto Cajal (CSIC), Pozuelo de Alarcón, 28223, Spain
[*]bmihaljevic@fi.upm.es

ABSTRACT

1 Morphometrics' conditional distributions

Branch type	Species	Tortuosity	Diameter	Distance	Length	RBA	RTA	Taper
non-terminal	human	0.00	0.00	0.00	0.00	0.16	0.03	0.00
non-terminal	mouse	0.00	0.00	0.00	0.00	0.53	0.08	0.00
terminal	human	0.00	0.08	0.00	0.04			0.00
terminal	mouse	0.00	0.32	0.03	0.92			0.00

Table 1. p-values for the Kolmogorov-Smirnov test of normality. Columns 1-2 indicate the groups and columns 3-9 show the p-values. RBA = remote bifurcation angle. RTA = remote tilt angle. Missing entries indicate that there were no observations for a given group.

Branch type	Species	BO	Tortuosity	Diameter	Distance	Length	RBA	RTA	Taper
non-terminal	human	1	0.00	0.00	0.96	0.00	0.25	0.35	0.02
non-terminal	human	2	0.00	0.01	0.06	0.00	0.30	0.15	0.10
non-terminal	human	3	0.00	0.07	0.26	0.00	0.31	0.51	0.08
non-terminal	human	4	0.00	0.25	0.57	0.00	0.33	0.14	0.11
non-terminal	human	5	0.51	0.29	0.88	0.16	0.60	0.76	0.33
non-terminal	mouse	1	0.20	0.12	0.90	0.01	0.77	0.77	0.01
non-terminal	mouse	2	0.48	0.02	0.09	0.00	0.60	0.29	0.21
non-terminal	mouse	3	0.05	0.19	0.01	0.00	0.59	0.82	0.36
non-terminal	mouse	4	0.04	0.14	0.36	0.04	0.94	0.75	0.71
non-terminal	mouse	5	0.29	0.13	0.45	0.40	0.66	0.99	0.71
terminal	human	2	0.08	0.36	0.25	0.56			0.01
terminal	human	3	0.00	0.28	0.00	0.31			0.00
terminal	human	4	0.00	0.30	0.00	0.40			0.00
terminal	human	5	0.00	0.83	0.00	0.58			0.17
terminal	mouse	2	0.09	0.76	0.52	0.42			0.18
terminal	mouse	3	0.71	0.20	0.08	0.85			0.01
terminal	mouse	4	0.11	0.64	0.14	0.86			0.01
terminal	mouse	5	0.04	0.36	0.79	0.95			0.09

Table 2. p-values for the Kolmogorov-Smirnov test of normality. Columns 1-3 indicate the groups and columns 4-10 show the p-values. RBA = remote bifurcation angle. RTA = remote tilt angle. Missing entries indicate that there were no observations for a given group.

2 Non-terminal branches: correlations

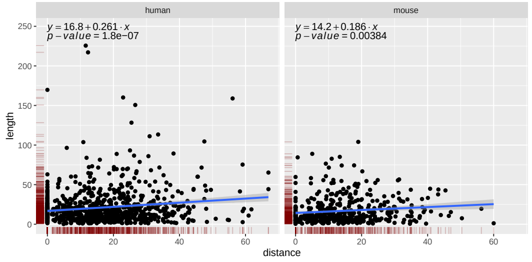

(a)

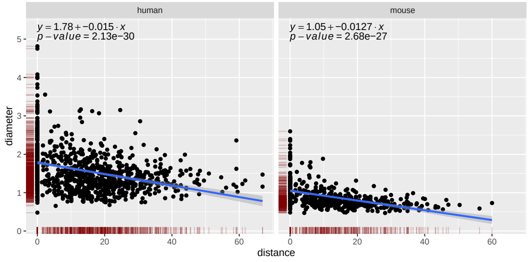

(b)

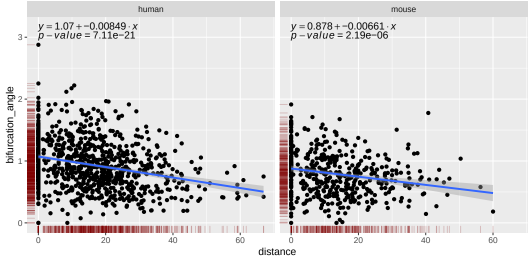

(c)

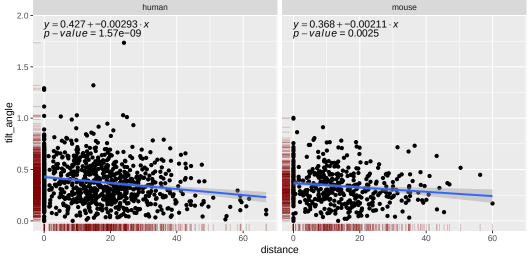

(d)

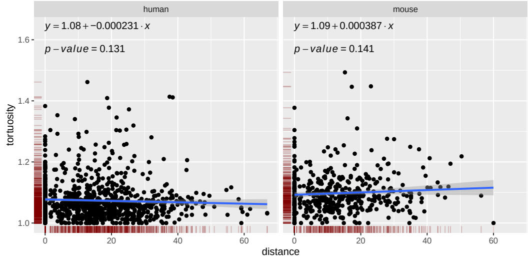

(e)

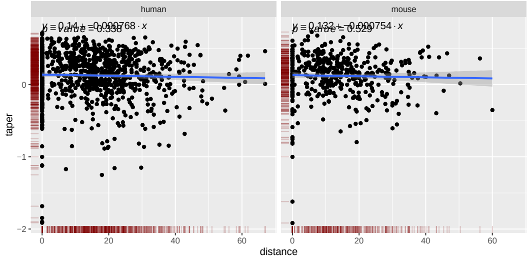

(f)

Figure 1. Non-terminal branches. Linear correlation of `distance` and `length`, `diameter`, `bifurcation_angle`, `tilt_angle`, `tortuosity` and `taper`. The sign of the correlation coefficient was the same for both species in all cases, with the regression lines roughly parallel.

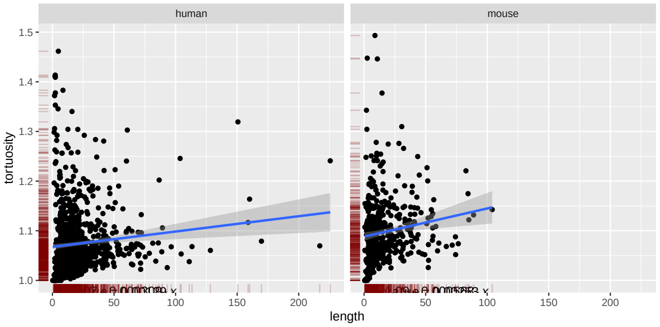

Figure 2. Non-terminal branches. `tortuosity` as a function of `length`.

3 Complete and incomplete branches

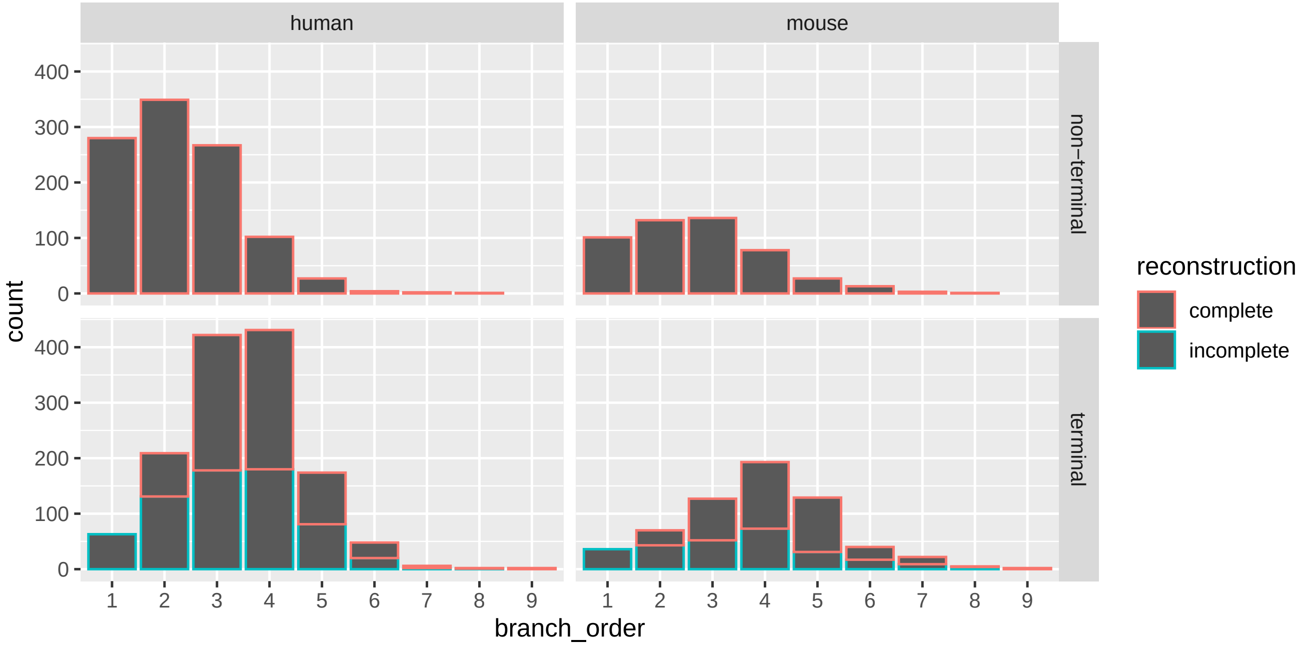

Figure 3. Complete and incomplete branch frequencies per species, branch type, and branching order.

4 Non-terminal branches: inter-species differences when conditioning on branch order

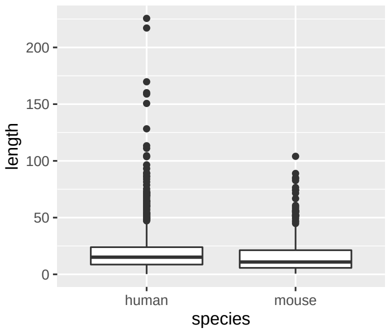

(a)

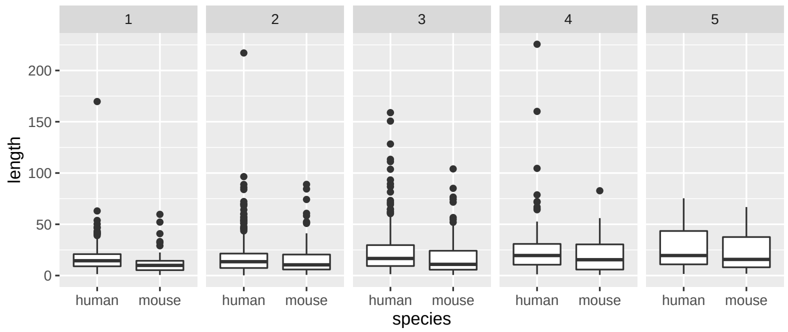

(b)

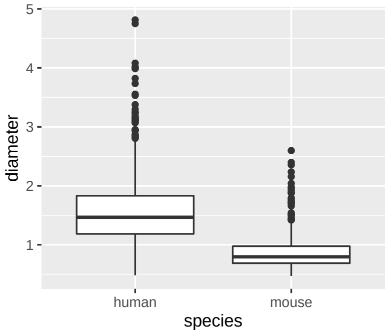

(c)

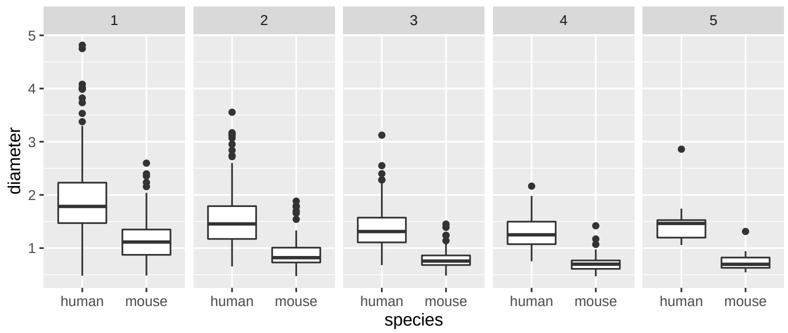

(d)

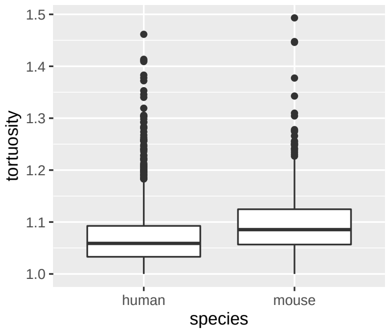

(e)

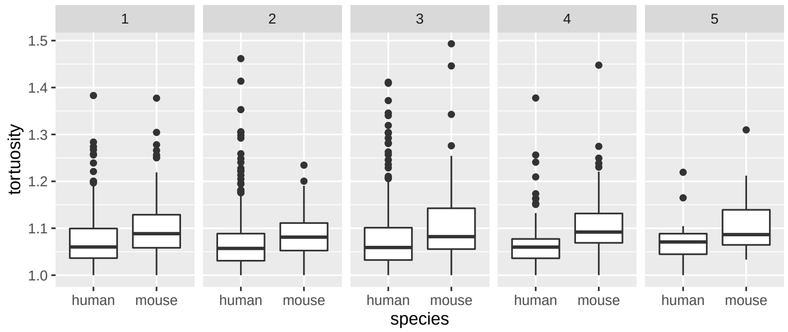

(f)

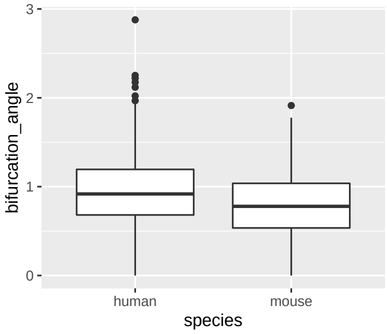

(g)

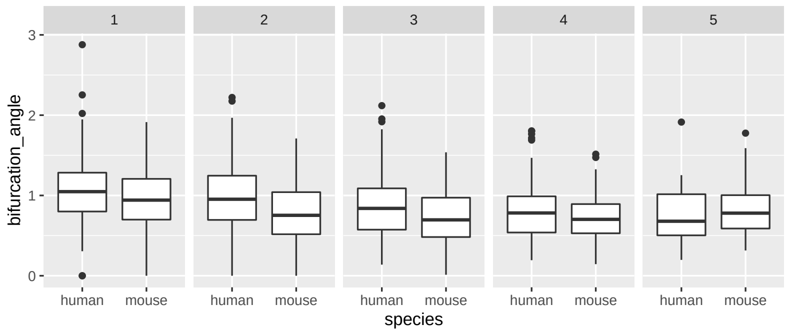

(h)

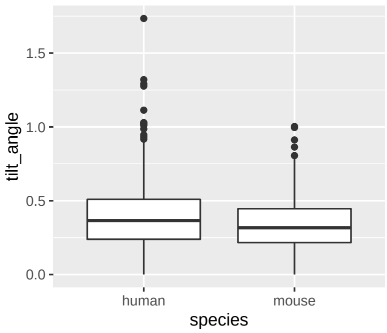

(i)

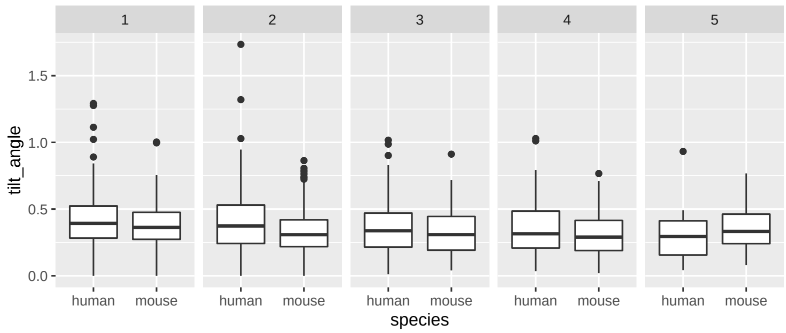

(j)

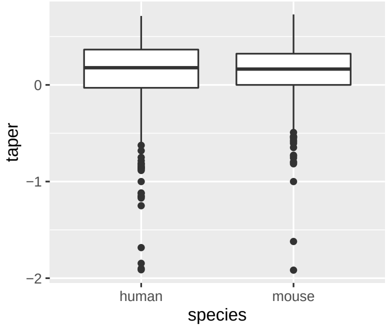

(k)

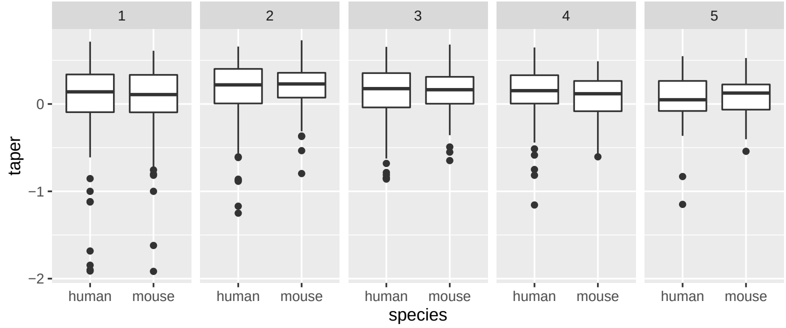

(l)

Figure 4. Differences in magnitude of variables of non-terminal branches between the species. (a)-(b) `length`: Marginal KW p-value 1.793e-07. KW p-values 6.71e-07, 0.08872, 0.0004447, 0.05116, and 0.4398 for branch orders 1 to 5, respectively; (c)-(d) `diameter`: Marginal KW p-value 6.408687e-136. KW p-values below 2.94e-09 for every branch order; (e)-(f) `tortuosity` : Marginal KW p-value 0004082. KW p-values 2.27e-06, 1.562e-05, 8.54e-07, 0.05703 for branch orders 1 to 5, respectively; (g)-(h) `bifurcation_angle`: Marginal KW p-value 6.841e-13. KW p-values 0.02463, 1.653e-06, 0.0004749, 0.142, and 0.5848 for branch orders 1 to 5, respectively; (i)-(j) `tilt_angle`: Marginal KW p-value 0.000121. KW p-values 0.1185, 0.003194, 0.3144, 0.1535, and 0.3001 for branch orders 1 to 5, respectively; (k)-(l) `taper` Marginal KW p-value 0.296145. KW p-values 0.597, 0.8745, 0.8758, 0.1096, and 0.5848 for branch orders 1 to 5, respectively.

5 Terminal branches: Hillman's taper rate

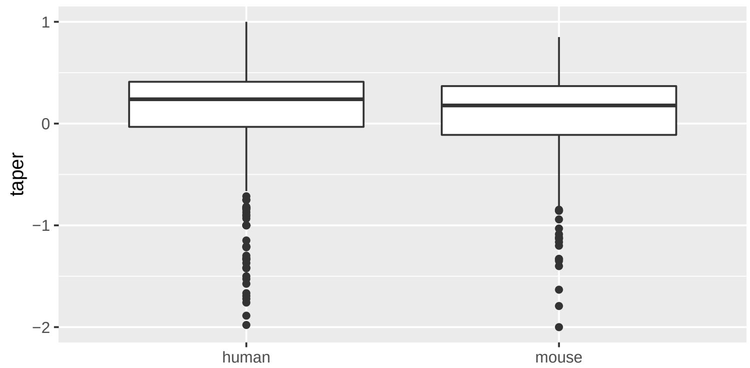

Figure 5. Differences in magnitude of `taper` for terminal branches between the species. KW p-value 0.026.

6 All branches: Hillman's taper rate

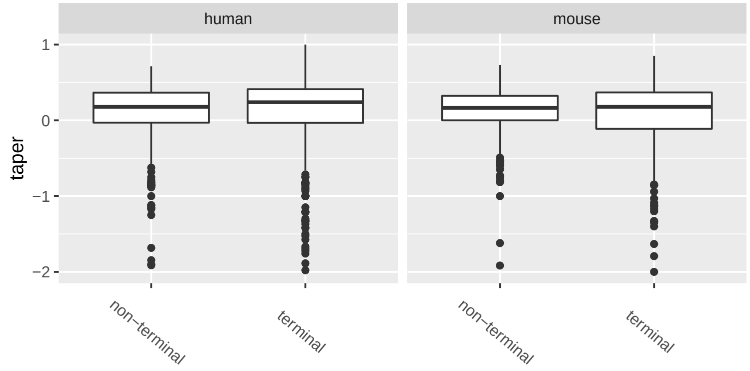

Figure 6. Differences in the magnitude of `taper` between terminal and non-terminal branches across species. KW p-values 0.018 and 0.810 for the human and mouse, respectively.
